# Social Welfare Centers Protect Outpatients with Mood Disorders from Risk of Hospital Admission

**DOI:** 10.1371/journal.pone.0146754

**Published:** 2016-01-08

**Authors:** Kyu-Tae Han, Suk Yong Jang, Sohee Park, Kyung Hee Cho, Ki-Bong Yoo, Young Choi, Eun-Cheol Park

**Affiliations:** 1 Department of Public Health, Graduate School, Yonsei University, Seoul, Republic of Korea; 2 Institute of Health Services Research, Yonsei University College of Medicine, Seoul, Republic of Korea; 3 Department of Biostatistics, Graduate School of Public Health, Yonsei University, Seoul, Republic of Korea; 4 Department of Hospital Management, Eulji University, Seongnam, Republic of Korea; 5 Department of Preventive Medicine, Yonsei University College of Medicine, Seoul, Republic of Korea; National Institute of Child Health and Human Development, UNITED STATES

## Abstract

**Background:**

South Korea faces difficulties in the management of mental disorders, and those difficulties are expected to gradually worsen. Therefore, we analyzed the relationship between social welfare centers and hospital admission after outpatient treatment for mood disorders.

**Methods:**

We used data from the National Health Insurance Service National Sample Cohort 2002–2013, which included all medical claims filed for the 50,160 patients who were newly diagnosed with a mood disorder among the 1,025,340 individuals in a nationally representative sample. We performed a logistic regression analysis using generalized estimating equation (GEE) models to examine the relationship between social welfare centers and hospital admission after outpatient treatment for mood disorders (ICD-10: F3).

**Results:**

There was a 3.9% admission rate among a total of 99,533 person-years. Outpatients who lived in regions with more social welfare centers were less likely to be admitted to a hospital (per increase of five social welfare centers per 100,000 people; OR: 0.958; 95% CI: 0.919–0.999). Social welfare centers had an especially strong protective effect on patients with relatively mild mood disorders and those who were vulnerable to medical expenditures.

**Conclusions:**

Considering the protective role of social welfare centers in managing patients with mood disorders, health-policy makers need to consider strategies for activating mental healthcare.

## Background

Mood disorders are mental disorders characterized by dramatic changes in or extremes of mood. They are classified into three types: depression, bipolar disorder, and anxiety disorder. Depression is defined as feelings of extreme sadness and hopelessness. Bipolar disorder is a mental illness marked by extreme shifts in mood ranging from a manic state to a depressive state. Anxiety disorders are characterized by feelings of nervousness, anxiety, and even fear. People with these diseases could experience problems in daily life and a reduction in quality of life [1].

Recently, mood disorders have been common in South Korea. The Epidemiological Survey of Mental Disorders in Korea showed that the lifetime prevalence of mood disorders has gradually increased in Korea in recent years (2001: 4.6%; 2006: 6.2%; 2011: 7.5%) [2]. The statistics of the Health Insurance Review & Assessment Services indicate that healthcare expenditures for mood disorders have rapidly increased in South Korea (2009: 263 billon KRW; 2013: 319 billion KRW) [3]. In addition, previous studies suggest that severe cases of mood disorders can progress to suicidal ideation or suicide attempts [4, 5]. Given that suicide management is relatively poor in South Korea compared with that in other Organization for Economic Cooperation and Development (OECD) countries, new ways of effectively managing mood disorders in South Korea are needed [6].

Although there are several treatments for mood disorders, the provision of those treatments is inadequate in South Korea, because South Koreans tend to avoid visiting mental healthcare centers, and mental healthcare resources are lacking in South Korea compared with those in other countries [2, 6]. Previous studies suggest that introducing new programs or expanding healthcare resources for mental healthcare might be effective [7, 8]. It is difficult in practice, however, to apply national programs because of economic issues including limitations on resources [9]. Therefore, an effective strategy is needed that considers several issues. In South Korea, where difficulties in the management of mental health problems have recently arisen, an emerging idea is the importance of the management of social and environmental factors, which may have an effect on mental health.

The South Korean government has designated social welfare centers to provide social welfare services and assist residents in the community. Based on the Social Service Act, social welfare centers have been operated with government funding and donations from sponsors, and people who wish to receive social support can freely use social welfare centers. These facilities provide support for people facing social problems, including those with disabilities, children, homeless people, elderly populations, and people with problems related to mental health. In cases of caring for people with mental problems, social workers generally provide social services as case management. For severe cases, social workers can cooperate with experts such as psychiatrists in the management of such people [10]. By Statistics Korea, the number of social welfare centers has continuously increased since the turn of the century (2003: 2.8 per 100,000 individuals; 2012: 13.0 per 100,000 individuals) [11].

Considering the increase in availability of social welfare centers and the their role towards those with social problems, social welfare centers might be having a positive effect on the mental health of residents, in particular, individuals with mental disorder; however, there are few studies of the relationship between social welfare services and mental healthcare in South Korea. Therefore, it is worthwhile to investigate the role of social welfare centers in providing mental healthcare to the community. We assumed that people who lived in regions with a superior socio-environmental atmosphere (i.e., more social welfare facilities) would be able to better manage their mental health. We analyzed the relationship between social welfare centers and psychiatric admission due to mood disorder among individuals who had received outpatient treatment for a mood disorder.

## Methods

### Study Population

We used data from the National Health Insurance Service National Sample Cohort 2002–2013, which were released by the Korean National Health Insurance Service (KNHIS) in 2014. The data comprise a nationally representative random sample of 1,025,340 individuals, approximately 2.2% of the entire population in the KNHIS in 2002. The data were produced by using probabilistic sampling to represent an individual’s total annual medical expenses within each of 1476 strata defined by age, sex, eligibility status (employed or self-employed), and income level (20 quantiles for each eligibility status plus medical-aid beneficiary) combinations via proportional allocation from the 46,605,433 Korean residents recorded in 2002 [12]. The database includes all medical claims filed from January 2002 to December 2013. To analyze the relationship between social welfare centers and the admission of individuals who had made previous outpatient visits due to a mood disorder, we included in the study only patients who were newly diagnosed with a mood disorder (ICD-10: F3) during outpatient care after 2003 (50,160 patients). We determined which of those patients were hospitalized during the study period due to the diagnosed mood disorder. Follow-up was performed every year, and the final sample used in our study included 99,533 person-years during 2003–2013. Regional characteristics were determined from the ‘e-provincial indicators’ published by Statistics Korea, which contained the regional demographic structures for the 253 basic administrative Si-Gun-Gu (city-county-ward) districts of South Korea. To consider the regional characteristics of the community where each patient lived, we classified the data based on the Si-Gun-Gu information.

### Variables

The outcome variable was psychiatric admission due to mood disorder in patients who had experience of previous outpatient visits due to mood disorder. We identified the date of each patient`s first outpatient visit during the study period, and we followed each patient after the date of the first outpatient visit. If an outpatient with a mood disorder was hospitalized due to the mood disorder in the same year, we considered the patient to have been admitted. We considered admission to reflect a worsening in the status of the patient.

The primary independent variable in relation to the psychiatric admission due to the mood disorder of outpatients was the number of social welfare centers per 100,000 residents in the communities where the patients lived. The social welfare centers play a role in providing social and welfare services to residents in each community. We hypothesized that the number of social welfare centers in each community is associated with the rate of hospital admission for a mood disorder among individuals in the community who had previously received outpatient treatment for the same mood disorder. We adjusted the data for patient-level and regional-level variables when analyzing the relationship between social welfare centers and the admission of outpatients. The patient-level variables included in the analyses were: sex, age, income, type of insurance coverage, year, presence of a mental disability, experience of pre-hospitalization after registration as an outpatient, and days of drug treatment per year. Age was categorized as <30 years, 30–39 years, 40–49 years, 50–59 years, 60–69 years, or >70 years. Income level was categorized as one of quintiles based on mean household income [13]. The types of insurance coverage were categorized as medical aid, KNHIS, employee insured, or KNHIS, self-employed insured based on the criteria of the KNHIS. Those with KNHIS, employee insured included workers and employers in all workplaces, public officials and private school employees, continuously insured persons, and daily-paid workers at construction sites. Beneficiaries of KNHIS, employee insured included spouses, descendants, brothers and sisters, and direct lineal ascendants. People with KNHIS, employee insured paid a regular portion (about 7%) of their average salary in contribution payments, the rates of which changed every year. The KNHIS, self-employed insured category included people excluded from the category of beneficiaries of KNHIS, employee insured. Their contribution amount was set by taking into account their income, property, living standard, and rate of participation in economic activities. Beneficiaries of medical aid were defined as patients with an income below the government-defined poverty level, or a disability; hence, this group was provided with free inpatient and outpatient care using government funds. Therefore, the type of insurance coverage represented the socio-economic status of each inpatient [14]. Individuals with severe mental health problems lasting more than 1 year were considered to have a mental disability, even if they were provided optimal treatment by doctors through the prequalification system [15]. We also included experience of pre-hospitalization and days of drug treatment per year to reflect the severity of disease in each patient [16]. Experience of pre-hospitalization was defined as an experience of hospitalization during the previous 1 year after baseline year of first diagnosis for mood disorder. This variable was designed to reflect the severity of illness in each patient, which could indicate the risk of subsequent hospitalization. Days of drug treatment per year was defined as the sum of the days of drug treatment for the mood disorder during each year.

The regional variables were the region type, population size, proportion of elderly population, number of cultural facilities, number of medical facilities, gross regional domestic product per population (GRDP), and financial independence rate of local government. The region types were metropolitan and others. The population size was defined as the total number of residents in each community, and the proportion of elderly population was defined as the number of elderly individuals among the total population of the community. The GRDP as value added on the production side was used as an indicator of how much value was added to economic activities in each region. The financial independence rate of the local government is an index of the finance utilization capacity of a local government with independent discretionary power [17, 18]. This indicator was calculated as follows: (local taxes + non tax revenue) / budgets of local government ×100.

### Statistical analyses

We first examined the frequencies and percentages of each categorical variable at the baseline of each patient and performed χ^2^ tests for the distribution of person-years by each variable during the study period. To compare the average values and standard deviations of the continuous variables, we examined the mean and standard deviation of each continuous variable at the baseline and performed an analysis of variance (ANOVA) for each variable during the study period. Analyses were performed for both the patient-level and the regional-level variables. Next, to examine associations with risk of hospitalization in outpatients due to mood disorders, the method of analysis used in this study was logistic regression using generalized estimating equations (GEE). Generalized estimating equation (GEE) models with link logit that included both patient- and region-level variables were analyzed, as the data used in this study were hierarchically structured and had binary outcome variables [19]. This GEE model assumed proper distributions for each hospitalization case while taking into account the correlation among individuals within the region. In this study, the correlation structure was modeled as an exchangeable correlation structure. The goodness of fit for the GEE model was assessed using the quasi-likelihood under the independence criterion (QIC). The lower value for QIC indicated the goodness of fit. Additionally, subgroup analyses were performed according to age group, type of insurance coverage, experience of pre-hospitalization, experience of drug treatment, region type, and number of medical facilities in each region. All statistical analyses were performed using SAS statistical software version 9.2.

### Ethics Statement

The data used in our study was consists to details for patient`s utilization of healthcare. This study was approved by the Institutional Review Board, Yonsei University Graduate School of Public Health (2014–239). And, this study was not included informed consents from the patients, because the patient`s information was anonymized and unidentified prior to analysis.

## Results

The data used in the analysis included 50,160 patients at baseline and 99,533 person-years during the study period.

Table 1 shows the general baseline characteristics including the patient-level and regional-level variables of the study population. The average number of social welfare centers was 6.3 per 100,000 community residents. The average proportion of elderly residents was 10.2% at baseline. The average number of medical facilities was 9.2 per 100,000 community residents. Females were more common than males (females: 66.6%). The average financial independence rate of the local government was 62.2%. There were generally more individuals in the lower age group than in the older age groups (<30 years: 21.8%; 30–39 years: 16.5%; 40–49 years: 18.3%; 50–59 years: 16.7%; 60–69 years: 14.0%; and >70 years: 12.7%). The distribution by income was as follows: 15.5% group 1 (low), 14.7% group 2, 16.9% group 3, 21.4% group 4, and 31.5% group 5 (high). KNHIS employee insured was the most common type of insurance coverage (medical aid: 1.4%; KNHIS self-employed insured: 40.2%; KNHIS employee insured: 58.4%). Patients with mental disabilities made up 0.3% of the total study population. The average number of drug treatment days at baseline was 3.7 days. The average number of follow-up visits was 2.0 during the study period.

**Table 1:** Characteristics of study population at baseline.

Variables	N/ Mean	%/ SD
**Regional variables**		
**Number of social welfare centers per 100,000 residents**	6.3	6.0
**Number of cultural centers per 100,000 residents**	3.5	3.9
**Number of medical centers per 1,000 residents**	9.2	5.0
**Population size**	405,464.5	248994.3
**Proportion of elderly population (%)**	10.2	4.9
**Gross regional domestic product per population (million KRW)**	22.3	7.3
**Financial independence rate of local government**	62.2	23.5
**Region**		
Metropolitan	23,015	45.9
Others	27,145	54.1
**Individual variables**		
**Sex**		
Male	16,768	33.4
Female	33,392	66.6
**Age (Years)**		
<30	10,942	21.8
30–39	8,261	16.5
40–49	9,197	18.3
50–59	8,374	16.7
60–69	7,005	14.0
>70	6,381	12.7
**Income**		
Group 1 (low)	7,764	15.5
Group 2	7,349	14.7
Group 3	8,483	16.9
Group 4	10,756	21.4
Group 5 (high)	15,808	31.5
**Types of insurance coverage**		
Medical aid	727	1.4
KNHIS, self-employed insured	20,152	40.2
KNHIS, employee insured	29,281	58.4
**Index year**		
2003	4,827	9.6
2004	4,780	9.5
2005	4,962	9.9
2006	4,702	9.4
2007	4,961	9.9
2008	4,350	8.7
2009	4,320	8.6
2010	4,268	8.5
2011	4,324	8.6
2012	4,612	9.2
2013	4,054	8.1
**Mental disability**		
Yes	156	0.3
No	50,004	99.7
**Drug treatment days per year**	3.7	20.3
**Average time of follow up**	2.0	1.8
**Total**	50,160	100.0

Table 2 shows the associations between the patient-level and regional variables and psychiatric admission due to mood disorder of outpatients during the study period. There was a 3.9% admission rate among the total 99,533 person-years. The average regional population size for the patients who were admitted was smaller than that for the patients who were not admitted. On the other hand, the average proportion of elderly residents in the community was higher for patients who were admitted than for the patients who were not admitted. The average numbers of cultural and medical facilities were higher for patients who were admitted than for those who were not admitted. The average rate of financial independence of the local government was higher for patients who were not admitted. Males were more frequently hospitalized than females (males: 4.6%; females: 3.6%). Individuals in the lower income group were more frequently hospitalized than those in other income groups (low: 4.5% group 1, 4.0% group 2, 3.9% group 3, and 3.8% group 4; high: 3.8% group 5). Beneficiaries of medical aid were more frequently hospitalized than individuals with other types of insurance (medical aid: 10.0%; KNHIS self-employed insured: 4.2%; KNHIS employee insured: 3.6%). Patients with mental disabilities or experiences of pre-hospitalization were more frequently hospitalized than patients without those factors (mental disability: 21.5%, no mental disability: 3.8%; pre-hospitalization: 33.0%, no pre-hospitalization: 3.2%). Patients who were admitted had fewer drug treatment days per year on average (4.0 days) than patients who were not admitted (6.8 days).

**Table 2:** Characteristics of study population by hospitalization during study period.

Variables	Hospitalization (N = 99,533)				P-value
	Yes		No		
	N/ Mean	%/ SD	N/ Mean	%/ SD	
**Regional variables**					
**Number of social welfare centers per 100,000 residents**	7.0	5.9	7.0	6.3	0.7175
**Number of cultural centers per 100,000 residents**	3.9	4.6	3.8	4.2	0.0337
**Number of medical centers per 1,000 residents**	9.9	5.4	9.5	5.1	<.0001
**Population size**	389,740.0	246,039.6	406,500.4	249,267.7	<.0001
**Proportion of elderly population (%)**	10.9	5.3	10.6	5.0	0.0002
**Gross regional domestic product per population (million KRW)**	23.4	7.6	23.4	7.4	0.7299
**Financial independence rate of local government**	59.5	24.0	62.4	23.5	<.0001
**Region**					
Metropolitan	1,765	3.8	44,347	96.2	0.1012
Others	2,153	4.0	51,268	96.0	
**Individual variables**					
**Sex**					
Male	1,479	4.6	30,955	95.4	<.0001
Female	2,439	3.6	64,660	96.4	
**Age (Years)**					
<30	758	4.5	16,001	95.5	<.0001
30–39	669	4.6	14,016	95.4	
40–49	710	4.0	17,176	96.0	
50–59	659	3.6	17,748	96.4	
60–69	520	3.3	15,377	96.7	
>70	602	3.8	15,297	96.2	
**Income**					
Group 1 (low)	683	4.5	14,584	95.5	0.0026
Group 2	570	4.0	13,605	96.0	
Group 3	642	3.9	15,656	96.1	
Group 4	789	3.8	20,105	96.2	
Group 5 (high)	1,234	3.8	31,665	96.2	
**Types of insurance coverage**					
Medical aid	124	10.0	1,121	90.0	<.0001
KNHIS, self-employed insured	1,636	4.2	36,863	95.8	
KNHIS, employee insured	2,158	3.6	57,631	96.4	
**Year**					
2003	207	4.3	4,620	95.7	0.1251
2004	261	4.0	6,276	96.0	
2005	279	3.5	7,614	96.5	
2006	337	4.0	8,069	96.0	
2007	398	4.2	9,086	95.8	
2008	382	4.1	8,832	95.9	
2009	386	4.0	9,323	96.0	
2010	387	3.9	9,614	96.1	
2011	446	4.2	10,177	95.8	
2012	430	3.7	11,055	96.3	
2013	405	3.6	10,949	96.4	
**Mental disability**					
Yes	154	21.5	561	78.5	<.0001
No	3,764	3.8	95,054	96.2	
**Experience of pre-hospitalization**					
Yes	786	33.0	1,598	67.0	<.0001
No	3,132	3.2	94,017	96.8	
**Drug treatment days per year**	4.0	21.3	6.8	34.3	<.0001
**Total**	3,918	3.9	95,615	96.1	

Table 3 shows the results of the logistic regression analysis using a generalized estimating equation model to investigate the relationship between social welfare centers and the psychiatric admission due to mood disorder in outpatients. The number of social welfare centers in a region was inversely associated with the risk of psychiatric admission (per increase of five social welfare centers per 100,000 residents: OR: 0.958; 95% CI: 0.919–0.999). The financial independence rate of the local government was inversely associated with psychiatric admission due to mood disorder (per increase of 10% financial independence rate of local government; OR: 0.939; 95% CI: 0.917–0.962). On the other hand, the number of medical centers in a region was negatively associated with the risk of admission due to mood disorder. Male patients had a higher risk of admission than female patients (OR: 1.270; 95% CI: 1.174–1.373). Moreover, patients who were beneficiaries of medical aid or who were KNHIS, self-employed insured were more likely to be admitted due to a mood disorder than patients who were KNHIS, employee insured (medical aid, OR: 2.336, 95% CI: 1.851–2.949; KNHIS self-employed insured, OR: 1.174, 95% CI: 1.089–1.265; ref: KNHIS employee insured). In addition, patients who had a mental disability or experience of pre-hospitalization were more likely to be admitted due to a mood disorder than individuals who did not have those factors (mental disability, OR: 3.316, 95% CI: 2.400–4.581; experience of pre-hospitalization, OR: 2.929, 95% CI: 2.332–3.677).

**Table 3:** Results of logistic regression analysis using GEE model for individual-level and regional-level variables.

Variables	Hospitalization			
	OR	95% CI		P-value
**Regional variables**				
**Number of social welfare centers per 100,000 residents (per increase of five centers)**	0.958	0.919	0.999	0.0419
**Number of cultural centers per 100,000 residents (per increase of five centers)**	1.001	0.941	1.063	0.9923
**Number of medical centers per 1,000 residents (per increase of five centers)**	1.036	0.996	1.077	0.0822
**Population size (per increase of 100,000 residents)**	0.997	0.975	1.020	0.7835
**Proportion of elderly population (per increase of 10%)**	1.049	0.927	1.188	0.4476
**Gross regional domestic product per population (million KRW)**	1.004	0.998	1.010	0.2054
**Financial independence rate of local government (per increase of 10%)**	0.939	0.917	0.962	< .0001
**Region**				
Metropolitan	1.108	0.992	1.238	0.0680
Others	1.000	-	-	
**Individual variables**				
**Sex**				
Male	1.270	1.174	1.373	<.0001
Female	1.000	-	-	
**Age (Years)**				
<30	1.000	-	-	
30–39	0.996	0.882	1.125	0.9512
40–49	0.891	0.790	1.004	0.0575
50–59	0.840	0.744	0.948	0.0048
60–69	0.767	0.673	0.874	<.0001
>70	0.914	0.803	1.041	0.1746
**Income**				
Group 1 (low)	1.008	0.897	1.132	0.8948
Group 2	1.013	0.903	1.136	0.8260
Group 3	0.997	0.893	1.113	0.9548
Group 4	0.983	0.889	1.088	0.7413
Group 5 (high)	1.000	-	-	
**Types of insurance coverage**				
Medical aid	2.336	1.851	2.949	<.0001
KNHIS, self-employed insured	1.174	1.089	1.265	<.0001
KNHIS, employee insured	1.000	-	-	
**Study year**	0.970	0.954	0.986	0.0003
**Mental disability**				
Yes	3.316	2.400	4.581	<.0001
No	1.000	-	-	
**Experience of pre-hospitalization**				
Yes	2.929	2.332	3.677	<.0001
No	1.000	-	-	
**Drug treatment days per year (per increase of 7 days)**	0.982	0.972	0.992	0.0002
**QIC (null model)**	33040.821			
**QIC (full model)**	31287.636			

We also performed subgroup analyses for logistic regression analysis using GEE models to investigate the relationship between social welfare centers and psychiatric admission due to mood disorder in outpatients by age group, type of insurance coverage, experience of pre-hospitalization or drug treatment, region, and number of social welfare centers in the region. In the subgroup analysis by age group, there were no statistically significant differences in the association between social welfare centers and risk of admission due to mood disorder. In the subgroup analysis by type of insurance coverage, a higher number of social welfare centers was inversely associated with outpatient admission due to mood disorder only among KNHIS, self-employed insured individuals (per increase of five social welfare centers per 100,000 residents; OR: 0.93; 95% CI: 0.882–1.00; p-value < 0.05). In the subgroup analysis by experience of pre-hospitalization, a higher number of social welfare centers was associated with a lower risk of admission due to mood disorder among the patients with no experience of pre-hospitalization (per increase of five social welfare centers per 100,000 residents; OR: 0.947; 95% CI: 0.909–0.988).

**Fig 1:**
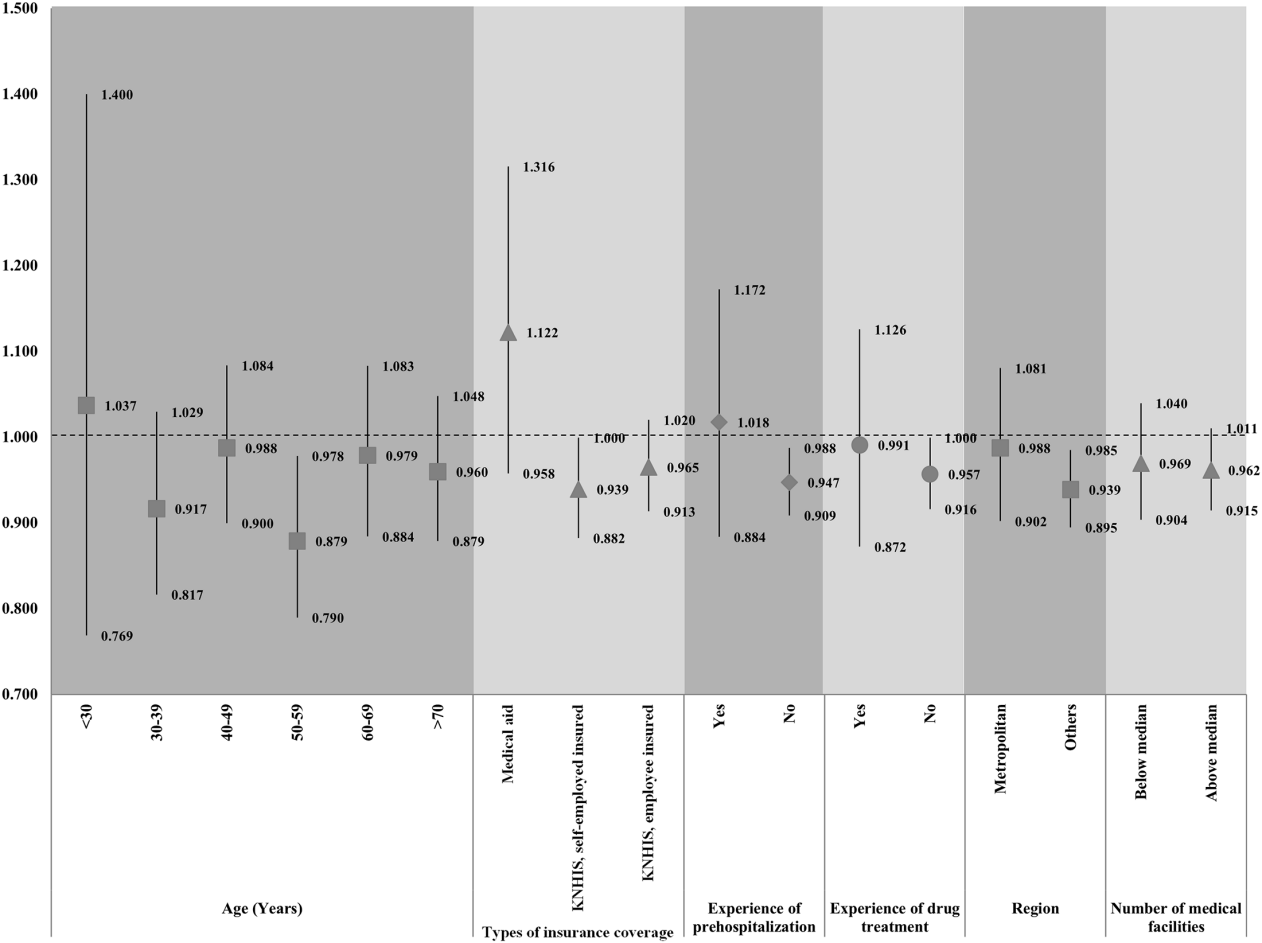
Results of subgroup analyses of multi-level models by age group, types of insurance coverage, experience of pre-hospitalization, drug treatment, region, and number of regional medical centers.

In addition, these associations were analyzed among the patients who did not receive drug treatment (per increase of five social welfare centers per 100,000 residents; OR: 0.957; 95% CI: 0.916–1.00; p-value < 0.05). In the subgroup analysis by regional-level variables such as region and number of medical centers, a higher number of social welfare centers was inversely associated with outpatient admission in non-metropolitan regions, although there were no statistically significant results based on the number of medical centers (non-metropolitan regions, per increase of five social welfare centers per 100,000 residents; OR: 0.939; 95% CI: 0.895–0.985; Fig 1).

## Discussion

Although many healthcare professionals have studied the effective management of mental health problems, there are many difficulties in applying effective changes to the healthcare system of South Korea, because most potential improvements require additional financial and human resources [9, 20]. To make a breakthrough, we assumed that regional social welfare centers might have a positive effect on the management of mental health problems, considering the Social Services Acts and the increasing number of social welfare centers in South Korea [11, 21]. We analyzed the relationship between social welfare centers and admission due to mood disorders. Our results show that patients living in regions with a higher number of social welfare centers were less likely to be admitted due to mood disorder. That finding suggests that social welfare centers help to prevent the deterioration of patients who are diagnosed with a mood disorder and receive outpatient treatment, because progression from outpatient care to hospital admission indicates a requirement for more intensive care due to worsening patient status [22, 23]. Although the South Korean government introduced community mental health centers after 1995 through the Mental Health Act as part of an effort to manage patients with mental disorders and improve the accessibility of mental healthcare for patients on a regional basis, those centers were not always effectively operated due to limited human and financial resources [24]. South Korea has had many difficulties in managing mental health problems, and using social welfare systems could be an effective way to improve mental health in South Korea [2, 25].

Our subgroup analysis showed that a higher number of regional social welfare centers was associated with a lower risk of psychiatric admission due to mood disorder among outpatients who had no experience of pre-hospitalization or drug treatment. These associations were also present among patients who were KNHIS, self-employed insured and among patients who lived in non-metropolitan areas. These findings suggest that regional social welfare centers had a greater positive effect on patients with mild mood disorders than on patients with more severe mood disorders. In addition, a higher number of social welfare centers had a greater protective effect on patients who were vulnerable to medical expenditures due to being KNHIS, self-employed insured. Therefore, managing mild mood disorders by creating social welfare centers could be an effective way to prevent the status of patients with mood disorders from worsening, the increase in healthcare expenditures due to mental disorders, and the increase in suicidal problems in South Korea.

Our results suggest that health-policy makers and decision makers should consider providing more support to social welfare centers and establishing alternative ways to activate social welfare centers in South Korea. If there were sufficient human and financial resources for mental healthcare in South Korea, the clinical management of mental health problems would likely be the best way to improve the overall level of mental health. Considering the worsening status of mental health in South Korea, a review of effective, non-clinical alternatives is needed.

Our study has several strengths compared with previous studies. We used national sampling cohort data to identify the relationship between social welfare centers and the admission of outpatients for mood disorders. Therefore, the data used in this study are especially helpful for establishing evidence-based policy for mental healthcare. To our knowledge, this study is the first attempt to investigate the role of social welfare centers in mental healthcare in South Korea. Although many previous studies investigated factors such as social support or community mental health services, few studies looked at the role of social welfare centers in mental healthcare in South Korea [26, 27]. The results of our study suggest alternatives for the effective management of mental disorders by taking another point of view. We used the admission of outpatients due to mood disorders as the outcome variable. Mood disorders are common and are expected to become more prevalent in South Korea in the future, and our results can be used to improve the management of patients with mood disorders. Finally, we adjusted the data for experience of drug treatment and pre-hospitalization to provide a more detailed study. Thus, we could reflect the severity of the mood disorders experienced by the patients in our analyses [28, 29].

Our study has also some limitations. Based on previous studies of mental healthcare, factors such as job status, marital status, family history of mental disorders, and other factors are associated with mental health [30–32]. However, the relevant details were not included in the data, as the data used in this study had characteristics of health insurance claim data. Therefore, we were unable to consider all of the potential factors which could affect the deterioration of mental health. Next, we could not determine whether individual patients with mood disorders received services from social welfare centers to manage their mood disorder, because that information was not included in the data. Third, considering the tendency of South Koreans to avoid visits to mental healthcare centers and the use of mental healthcare resources, the prevalence of mood disorders might be underestimated in our study, because there might be unidentified patients with mood disorders in the nationally sampled data [33]. Fourth, we did not consider other types of treatment and comorbid psychiatric disease that might have been provided to patients with mood disorders, due to the limitations of the data. Finally, the outcome variables used in this study were defined as admission due to mood disorder in patients who had been diagnosed with mood disorder. However, based on previous studies, the diagnoses of psychiatric diseases were not stable from outpatient treatment to admission. Thus, it is possible that the measurement of the risk of hospitalization could have been underestimated in this study [34].

Despite these limitations, our findings suggest that social welfare centers play a protective role in the lives of patients with mood disorders, particularly those whose disorders are relatively mild. Given the difficulties related to mental health in South Korea and the emerging importance of the management of social and environmental factors that can affect mental health, these findings could be helpful in the management of the mental health of the overall population, from the perspective of public health. Although further studies using more detailed data will be needed in the future and the impact of social welfare centers on the management of patients with mood disorders did not play a sufficiently protective role compared to the medical treatment of patients, health policymakers and decision makers in mental healthcare should consider effective alternatives for activating the protective role of social welfare centers for patients with mood disorders.

## Conclusions

Our findings suggest that a higher number of social welfare centers is inversely associated with the risk of outpatient admission for mood disorders, particularly among patients with relatively mild disease. Considering the protective role of social welfare centers in the management of mood disorders, health-policy makers and decision makers need to consider strategies for activating social welfare centers for mental healthcare.

## References

[1] Sadock BJ, Sadock VA. Kaplan and Sadock's synopsis of psychiatry: Behavioral sciences/clinical psychiatry: Lippincott Williams & Wilkins; 2011.

[2] Ministry of Health & Welfare. The Epidemiological Survey of Mental Disorders in Korea. 2001, 2006, 2011.

[3] Health Insurance Review & Assessment Service. Statistics for diseases 2009–13.

[4] Nordström P, Åsberg M, Åberg‐Wistedt A, Nordin C. Attempted suicide predicts suicide risk in mood disorders. Acta Psychiatr Scand. 1995;92(5):345–50. 8619338 10.1111/j.1600-0447.1995.tb09595.x

[5] Rihmer Z. Suicide risk in mood disorders. Curr Opin Psychiatry. 2007;20(1):17–22. 17143077 10.1097/YCO.0b013e3280106868

[6] Organization for Economic Cooperation and Development. Health at a Glance 2013: OECD Indicators. 2013.

[7] Amaddeo F, Zambello F, Tansella M, Thornicroft G. Accessibility and pathways to psychiatric care in a community-based mental health system. Soc Psychiatry Psychiatr Epidemiol. 2001;36(10):500–7. 11768848 10.1007/s001270170015

[8] Rost K, Fortney J, Fischer E, Smith J. Use, quality, and outcomes of care for mental health: The rural perspective. Med Care Res Rev. 2002;59(3):231–65. 12205828 10.1177/1077558702059003001

[9] Saxena S, Thornicroft G, Knapp M, Whiteford H. Resources for mental health: scarcity, inequity, and inefficiency. Lancet. 2007;370(9590):878–89. 17804062 10.1016/S0140-6736(07)61239-2

[10] Ministry of Health & Welfare. Social Services Act. 1997.

[11] Statistics Korea. E-provincial indicators.

[12] Kim NH, Lee J, Kim TJ, Kim NH, Choi KM, Baik SH, et al Body Mass Index and Mortality in the General Population and in Subjects with Chronic Disease in Korea: A Nationwide Cohort Study (2002–2010). PLoS One. 2015;10(10):e0139924 10.1371/journal.pone.0139924 26462235 PMC4604086

[13] Gunnell DJ, Peters TJ, Kammerling RM, Brooks J. Relation between parasuicide, suicide, psychiatric admissions, and socioeconomic deprivation. BMJ. 1995;311(6999):226–30. 7627035 10.1136/bmj.311.6999.226 PMC2550279

[14] Song YJ. The South Korean health care system. JMAJ. 2009;52(3):206–9.

[15] Kim S-S, Chung Y, Perry MJ, Kawachi I, Subramanian S. Association between interpersonal trust, reciprocity, and depression in South Korea: a prospective analysis. PLoS One. 2012;7(1):e30602 10.1371/journal.pone.0030602 22279597 PMC3261209

[16] Powell J, Geddes J, HAWTON K, DEEKS J, GOLDACRE M. Suicide in psychiatric hospital in-patients Risk factors and their predictive power. Br J Psychiatry. 2000;176(3):266–72. 10755075 10.1192/bjp.176.3.266

[17] Kim K-M, Park D. Impacts of urban economic factors on private tutoring industry. Asia Pac Educ Rev. 2012;13(2):273–80.

[18] Hamano T, Fujisawa Y, Ishida Y, Subramanian S, Kawachi I, Shiwaku K. Social capital and mental health in Japan: a multilevel analysis. PLoS One. 2010;5(10):1–6. 10.1371/journal.pone.0013214 PMC2950857 20949091

[19] Hanley JA, Negassa A, Forrester JE. Statistical analysis of correlated data using generalized estimating equations: an orientation. Am J Epidemiol. 2003;157(4):364–75. 12578807 10.1093/aje/kwf215

[20] Dussault G, Dubois C-A. Human resources for health policies: a critical component in health policies. Hum Resour Health. 2003;1(1):1 12904254 10.1186/1478-4491-1-1 PMC166115

[21] Ministry of Health & Welfare. Social Services Act

[22] Ruth U, Freisleder FJ, Heinrich H. Outpatient emergency admissions to a child and adolescent psychiatry hospital, and following immediate hospitalization. Ger J Psychiatry. 2013;16:1–6.

[23] Swigar ME, Astrachan B, Levine MA, Mayfield V, Radovich C. Single and repeated admissions to a mental health center: demographic, clinical and use of service characteristics. Int J Soc Psychiatry. 1991;37(4):259–66. 1783504 10.1177/002076409103700405

[24] Ministry of Health & Welfare. Mental Health Act. 1995.

[25] Cho MJ, Lee JY, Kim B-S, Lee HW, Sohn JH. Prevalence of the major mental disorders among the Korean elderly. J Korean Med Sci. 2011;26(1):1–10. 10.3346/jkms.2011.26.1.1 21218022 PMC3012831

[26] Lee J-S, Koeske GF, Sales E. Social support buffering of acculturative stress: A study of mental health symptoms among Korean international students. Int J Intercult Relat. 2004;28(5):399–414.

[27] Taylor SE, Sherman DK, Kim HS, Jarcho J, Takagi K, Dunagan MS. Culture and social support: who seeks it and why? J Pers Soc Psychol. 2004;87(3):354 15382985 10.1037/0022-3514.87.3.354

[28] Curyto KJ, Johnson J, TenHave T, Mossey J, Knott K, Katz IR. Survival of hospitalized elderly patients with delirium: a prospective study. Am J Geriatr Psychiatry. 2001;9(2):141–7. 11316618

[29] Padgett D, Struening EL, Andrews H. Factors affecting the use of medical, mental health, alcohol, and drug treatment services by homeless adults. Med Care. 1990:805–21. 2402175 10.1097/00005650-199009000-00010

[30] Tyssen R, Vaglum P, Grønvold NT, Ekeberg Ø. The impact of job stress and working conditions on mental health problems among junior house officers. A nationwide Norwegian prospective cohort study. Med Educ. 2000;34(5):374–84. 10760123 10.1046/j.1365-2923.2000.00540.x

[31] Han K-T, Park E-C, Kim J-H, Kim SJ, Park S. Is marital status associated with quality of life? Health Qual Life Outcomes. 2014;12(1):109. 25104276 10.1186/s12955-014-0109-0 PMC4148557

[32] Use FAT. Substance Abuse & Mental Health Services Administration. 2010. 24354028

[33] Lee CK, Kwak YS, Yamamoto J, Rhee H, Kim YS, Han JH, et al Psychiatric epidemiology in Korea: Part I: Gender and age differences in Seoul. J Nerv Ment Dis. 1990;178(4):242–6. 2319232 10.1097/00005053-199004000-00004

[34] Baca-Garcia E, Perez-Rodriguez MM, Basurte-Villamor I, Del Moral ALF, Jimenez-Arriero MA, De Rivera JLG, et al Diagnostic stability of psychiatric disorders in clinical practice. Br J Psychiatry. 2007;190(3):210–6. 17329740 10.1192/bjp.bp.106.024026

